# Gender differences in externalizing and internalizing problems in Singaporean children and adolescents with attention-deficit/hyperactivity disorder

**DOI:** 10.1186/s13034-021-00356-8

**Published:** 2021-01-22

**Authors:** Tsz Wing Ivy Lau, Choon Guan Lim, Sanchalika Acharryya, Nikki Lim-Ashworth, Yi Ren Tan, Shuen Sheng Daniel Fung

**Affiliations:** 1 The Institute of Mental Health, Buangkok Green Medical Park 10 Buangkok View, Singapore, 539747 Singapore; 2 Duke-NUS Medical School, 8 College Road, Singapore, 169857 Singapore

**Keywords:** Attention-deficit/hyperactivity disorder, ADHD, Gender comparison, Internalizing, Externalizing, Asia, Children and adolescents

## Abstract

**Background:**

Studies on gender differences in attention-deficit/hyperactivity disorder (ADHD) comorbidities in the Asian populations have been limited and previous studies have shown inconclusive findings. Singapore is a city-state country in Southeast Asia with a population of 5.7 million. This study examined gender differences in internalizing and externalizing problems in Singaporean children and adolescents with ADHD. The plausible social factors underlying the gender differences were discussed.

**Methods:**

A total of 773 participants (aged 6 to 18, 88% males) newly diagnosed with ADHD were recruited from the largest public child and adolescent psychiatric center in Singapore. Their internalizing and externalizing problems were assessed using the Child Behavioral Checklist and Teacher’s Report Form by parents and teachers respectively. Demographics and relevant social factors were collected using parent questionnaires.

**Results:**

Females with ADHD were reported to have less delinquent and aggressive behavior but more depressive symptoms than their male counterparts, similar to findings in the Western literature. Gender remained a significant predictor of externalizing problem after controlling for other factors. Lower socioeconomic status and parental use of physical punishment were significant predictors of both internalizing and externalizing problems.

**Conclusions:**

Gender differences in ADHD comorbidities do exist in the Asian clinical population. The lack of externalizing symptoms in females with ADHD has made timely referral and diagnosis challenging. More research is needed in understanding the gender differences in ADHD and the biopsychosocial mechanism underlying the differences in order to improve the detection of ADHD in females.

## Background

Singapore is an island city-state country of about 710 km^2^ in Southeast Asia with a population of 5.7 million [1]. Attention-deficit/hyperactivity disorder (ADHD) is a neurodevelopmental disorder that has been reported to affect 3–7% of school-age children worldwide [2, 3]. In Singapore, a nationwide epidemiological study on the prevalence of ADHD is not available at the time of this publication. According to a school-based study sampling 2139 children in Singapore, 4.9% of children were reported to have clinically significant behavioral problems [4]. ADHD was noted to be the leading diagnosis at the clinics of the Department of Developmental Psychiatry (DDP) at the Institute of Mental Health (IMH) which has been the largest public service provider of such specialty care in the country [5]. Apart from IMH, child and adolescent psychiatric service is also available at other restructured public hospitals and private mental health services in Singapore. In addition to psychiatrists, there are also clinical psychologists, occupational therapists, medical social workers and school counsellors who play a significant role in child and adolescent mental health services.

It is well known that ADHD affects more males than females and the gender ratio differs between the clinical samples and community samples. In a meta-analysis of 97 studies with community samples, the male to female ratio was reported to be about 3:1 [6]. In the clinical samples, the male to female ratio varied from 5:1 to 16:1 [2, 7–11]. The discrepancy between the clinical and community samples has suggested under-identification of ADHD in girls, probably due to the gender differences in the ADHD symptoms and comorbidities [9, 12]. Although researchers have recognized gender as a significant moderator of ADHD presentation for more than two decades, research on gender comparisons in ADHD has been limited due to the lower prevalence of ADHD in females.

Before the change from ADHD ‘subtypes’ to ‘presentations’ in the fifth edition of the Diagnostic and Statistical Manual of Mental Disorders [13], past studies have shown that females were more likely to be diagnosed with the ‘predominantly inattentive subtype’ while males were more likely to be of the ‘predominantly hyperactive and impulsive subtype’ [9, 12, 14–16]. However, there were studies that did not observe any gender differences in inattention, hyperactivity and impulsivity which suggested that females and males with ADHD were similar, especially in the clinical samples [8, 9, 11]. By comparing performance in cognitive tests measuring processing speed, inhibition and working memory between genders, Arnett et al. [14] postulated that gender differences in ADHD severity could be partially moderated by certain cognitive endophenotypes, suggesting a role of genetics in the gender differences. Although Arnett et al. [14] reported that males with ADHD were rated as more inattentive than their female counterparts by parents and teachers, a meta-analysis of studies using the Continuous Performance Test reported no gender differences in inattention but males with ADHD were more impulsive than females with ADHD [17].

Other than differences in core symptoms of ADHD, it has been reported that males and females with ADHD displayed different comorbidity profiles. In general, males with ADHD presented with externalizing problems such as conduct disorder and oppositional defiant disorder while females with ADHD presented with more internalizing problems such as anxiety and depression [9, 12, 18–20]. Similar gender discrepancy was reported in adults with ADHD in a recent Norwegian population-based study [21]. In addition, female adolescents with ADHD have been shown to suffer more peer rejection, poorer perceived locus of control and were more likely to be admitted for psychiatric issues in adulthood than their male counterparts [22, 23].

In addition to gender, age is another factor that moderates ADHD symptoms and comorbidities. In a prospective study of 128 boys with ADHD, decline in ADHD symptoms, especially hyperactivity and impulsivity, was noted as they grew up [24]. However, inattention problem was shown to be more persistent [25]. Comorbidities of ADHD also vary with age. Early age of onset has been shown to be associated with more aggressive problems while a later age of onset was associated with more anxiety and depressive symptoms [26].

To date, most studies on gender differences on ADHD have been done in the western population. Not much is known about the gender differences in the developmental population in Asia. Due to cultural differences, the perception of hyperactivity, the stigma of ADHD and the threshold of seeking professional help may be different in the Asian population. The limited studies in Asia suggested no gender differences in terms of ADHD symptoms and comorbidities [8, 11]. However, the numbers of females included in these studies were limited with 10 females in the Korean study [8] and 21 females in the Taiwanese study [11], which may have limited the statistical power of the comparison. Studies with a representative sample of females are needed to clarify gender differences in the Asian context.

This study aimed to examine the distribution of externalizing and internalizing symptoms between males and females with ADHD as well as between predefined age groups using parent-reports and teacher-reports through an Asian clinical sample, with adequate representation from each gender. The primary hypothesis was that females with ADHD would present with fewer externalizing and more internalizing symptoms compared to males with ADHD. We also hypothesized that the gender differences might be further moderated by age. The secondary aim attempted to investigate the effects of age, socioeconomic status, use of physical punishment and aggression between parents on internalizing and externalizing problems in ADHD. It was hypothesized that younger age, lower socioeconomic status, use of physical punishment and aggression between parents would be associated with more externalizing problems and older age would be associated with more internalizing problems.

## Methods

### Participants and procedures

This was a retrospective study utilizing an existing database (reference number IMH/2005-0005) maintained by DDP at IMH. Children and adolescents with learning, emotional or behavioral problems were referred to DDP clinics by parents, schools or medical doctors [27]. The registered database included information collected from 773 children and adolescents, aged 6 to 18, who had their first consultations at DDP clinics during 2005 to 2007. They were newly diagnosed with ADHD by the attending psychiatrists using the International Classification of Diseases, Ninth Revision, Clinical Modification (ICD-9-CM) based on clinical assessment which included a comprehensive interview with the young person and family, a structured report from the teacher and a computerized test of attention.

### Measures

All data was collected as part of routine assessments at the first clinic consultation. Written consents to use the collected data for research purposes were obtained from parents at the first clinic visit. Parents completed the Child Behavior Checklist (CBCL 6-18) while a school teacher who was familiar with the participant completed the Teacher’s Report Form (TRF).

### CBCL and TRF

Both CBCL and TRF are commonly used behavioral measures describing a child’s behavioral, emotional, and social problems over the past 6 months [28]. The version of CBCL and TRF for aged 6 to 18 was used in this study. Both CBCL and TRF have been demonstrated to have good internal consistency and criterion validity in Singapore. The Cronbach alphas for CBCL internalizing problems and externalizing problems were found to be 0.89 and 0.91 respectively while the alphas for TRF internalizing and externalizing problems were 0.88 and 0.95 respectively in Singapore [27]. There are 113 items in total e.g. “Cries a lot”, “Lying or cheating”. The items in CBCL and TRF are largely identical except for some items which have been adapted for the specific context. The scoring is the same for both measures. For each item, the score ranges from 0 = not true, 1 = somewhat/sometimes true, to 2 = very/often true. There are eight syndrome scales, namely anxious/depressed, withdrawn/depressed, somatic complaints, social problems, thought problems, attention problems, rule-breaking behavior and aggressive behavior. The syndrome scale scores are the sum of certain relevant item scores and the scores for internalizing problems and externalizing problems are the sum of specific syndrome scale scores. In this study, the raw scores of the CBCL and TRF scales were compared between genders as the raw scores reflect a better spread of data and allow a more sensitive comparison for research purposes [28].

Apart from CBCL, parents completed a standardized questionnaire which was adapted from the Ontario Child Health Study [29]. The questionnaire covered demographics of the child and parents, child’s medical and psychiatric histories, family psychiatric history, stressful life events, family functioning and parenting style. To investigate the effect of certain social factors on internalizing and externalizing problems, relevant items of the questionnaire were extracted for analysis.

Parental education levels and whether they have experienced financial problems were extracted as proxy of socioeconomic status of the family. Their educational levels were categorized into two levels: secondary education or below and post-secondary education.

To study the relationship between physical punishment and internalizing and externalizing problems, two relevant items were extracted. They were whether parents (1) spanked the young persons or (2) hit them with an object. To facilitate multivariate analysis, parents’ responses were coded as 0 = no physical punishment in disciplining, 1 = spanked or hit with an object, and 2 = spanked and hit with an object.

Psychological aggression between spouses was assessed by asking parents whether these statements were true or not: “When you had disagreements with your spouse, you resolved it by (1) raising voices and yelling at each other; (2) refusing to talk; (3) insulting or swearing; (4) crying and (5) leaving the room to avoid continuing the argument”. These statements were extracted from the Conflict Tactics Scale which was designed to explore conflict and violence within a family [30]. The responses to these items were all ‘yes = 1 point’ or ‘no = 0 point’. The total score for psychological aggression was measured as the sum of the five items.

### Data analysis

Distribution of demographic and clinical characteristics of the study population by gender was summarized using appropriate statistical measures. Internalizing and externalizing problem scores and syndrome scale scores of CBCL and TRF were compared between males and females. To study the effect of age on the above scores, the subjects were dichotomized into two age groups, 6 to 12 years old and 13 to 18 years old for comparison. The cut-off at 12 years old was chosen because it is the typical age when Singaporean students are being promoted from primary education to secondary education with significant changes in school environment. Normality checking was done by visual inspection of data distribution and Kolmongorov–Smirmov and Shapiro–Wilk tests. Mann–Whitney test was used for between genders and between age groups comparison in view of non-normal distribution of data and uneven group sizes with unequal variance. Effect sizes were calculated using the formula *r* = *Z/√N*, where *N* = number of observations [31]. Multiple linear regression models were developed separately for internalizing and externalizing scores using gender, age, gender × age interaction and other variables as explanatory variables to identify their association with internalizing and externalizing problems. Assumptions of linear relationship, multivariate normality, non-multicollinearity and homoscedasticity were met. Analysis was performed at two sided 5% significance level using SPSS version 24. Missing values for any of the variables were excluded.

## Results

**Fig. 1:**
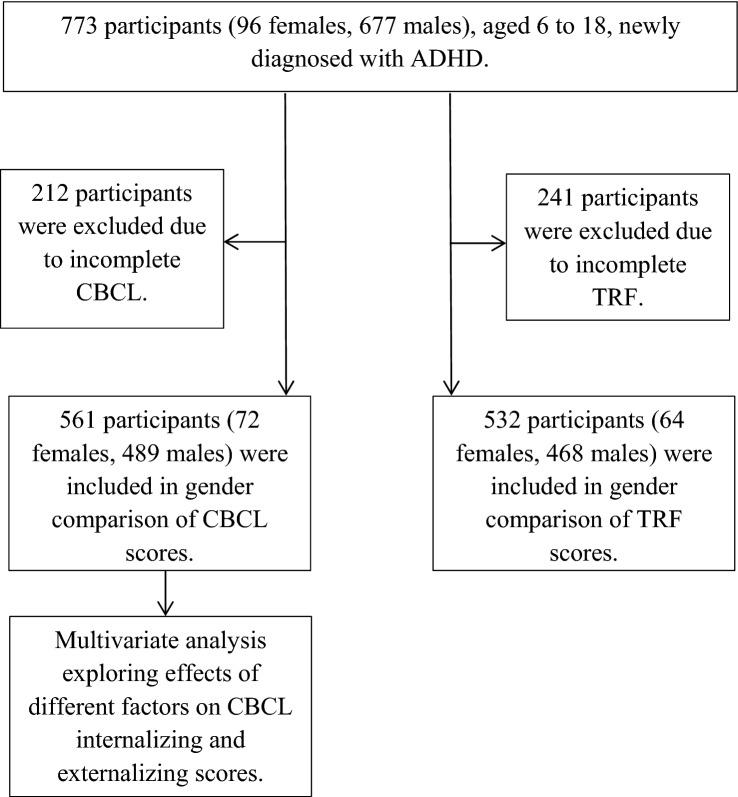
Flow diagram in analysis of CBCL and TRF scores

Among the 773 children and adolescents, there were 677 males and 96 females with a male to female ratio of 7:1. The gender ratio was consistent with those found in overseas and local clinical studies [11, 12, 32]. Out of the 773 participants, 561 (72%) participants, including 72 females and 489 males, have completed CBCL (Fig. 1). Among those with completed CBCL, 41.0% of mothers completed post-secondary education, compared to 32.3% of mothers in the group of incomplete CBCL, *χ*^2^ (1) = 4.95, *p* < 0.05. There were no statistically significant differences in fathers’ education level, ethnicity, type of housing, or parental occupation categories between the two groups.

The demographics and other characteristics of the participants were presented in Table 1. Eighty percent of them were Chinese, followed by Indian (7%), Malay (4%) and others (8%). Seventy-seven percent of them were staying at public housing estates. The ethnic ratio and the housing distribution of the sample were comparable to those of the general population [33]. This showed that our sample was largely representative of service users of child mental health services in Singapore. There were no statistically significant differences between genders in terms of their demographics, socioeconomic variables, psychological aggression between parents and parental use of physical punishment.

**Table 1:** Demographic and other variables by gender

	Female	Male
Number	96	677
Age range	6–15	6–18
Mean age (*SD*)	8.80 (1.88)	8.76 (2.18)
Ethnicity (%within gender)		
Chinese	81.8	80.0
Indian	9.1	7.0
Malay	4.5	4.2
Others	4.5	8.7
Mother’s education level (%within gender)		
Secondary education or below	52.7	61.4
Post-secondary or above	47.3	37.4
Others	0.0	1.2
Father’s education level (%within gender)		
Secondary education or below	48.3	54.6
Post-secondary or above	49.4	43.4
Others	2.3	2.1
Experienced financial problems	12.0	9.7
Psychological aggression between spouses (%within gender)		
Yell at spouse	36.9	41.3
Refuse to talk	40.5	35.4
Cry	15.5	9.5
Insult or swear	3.6	3.6
Threaten to hit or injure spouse	1.2	1.3
Physical punishment (%within gender)		
Spank with your hand	32.2	35.5
Cane or hit with an object	41.1	43.9
Shake or shove	2.2	1.9
History of family member(s) seeing a psychiatrist (%within gender)	8.0	12.9

CBCL internalizing problems, externalizing problems, syndrome scales and total problems were compared between genders using Mann–Whitney test (Table 2). As hypothesized, females with ADHD displayed less delinquent behavior (*U* = 14,042, *z* = − 2.79, *p* = 0.005, *r* = − 0.12), less aggressive behavior (*U* = 14,905, *z* = − 2.10, *p* = 0.035, *r* = − 0.09) but they were more withdrawn/depressed (*U* = 14,578, *z* = − 2.38, *p* = *0.0*17, *r* = − 0.10). Males with ADHD had a significantly higher score for externalizing problems (*U* = 14,747, *z* = − 2.23, *p* = 0.026, *r* = − 0.09) but the gender difference in internalizing scores was not statistically significant, *U* = 15,659, *z* = − 1.52, *p* = 0.129, *r* = − 0.06. There was no statistically significant gender difference in attention problems.

**Table 2:** CBCL and TRF syndrome scales, internalizing and externalizing problems by gender

	CBCL				TRF			
	Female (*n* = 72)	Male (*n* = 489)	*P*	Effect size *r*	Female (*n* = 64)	Male (*n* = 468)	*P*	Effect size *r*
	*Median (25th–75th percentile)*	*Median (25th–75th percentile)*			*Median (25th–75th percentile)*	*Median (25th–75th percentile)*		
Syndrome scales								
Withdrawn/depressed	4.0 (1.0–6.0)	2.0 (1.0–5.0)	0.017	− 0.10	1.5 (0.0–4.8)	2.0 (0.0–4.0)	0.819	− 0.01
Somatic complaints	2.0 (1.0–3.75)	2.0 (0.0–4.0)	0.416	− 0.03	0.0 (0.0–1.0)	0.0 (0.0–1.0)	0.930	− 0.00
Anxious/depressed	5.0 (2.0–9.8)	5.0 (2.0–8.0)	0.266	− 0.05	2.0 (0.0–5.0)	1.0 (0.0–4.0)	0.284	− 0.05
Social problems	5.5 (3.3–8.0)	5.0 (3.0–7.0)	0.159	− 0.06	2.0 (0.0–6.0)	3.5 (1.0–6.0)	0.030	− 0.09
Thought problems	2.0 (1.0–4.0)	2.0 (1.0–4.0)	0.669	− 0.02	0.0 (0.0–2.0)	1.0 (0.0–2.0)	0.034	− 0.09
Attention problems	10.0 (7.0–12.0)	10.0 (7.0–12.0)	0.913	− 0.00	15.5 (8.3–22.0)	20.0 (13.0–26.0)	0.000	− 0.15
Delinquent behavior	2.0 (1.0–5.0)	4.0 (2.0–6.0)	0.005	− 0.12	1.0 (0.0–3.0)	2.0 (1.0–4.0)	0.004	− 0.13
Aggressive behavior	12.0 (8.0–17.8)	14.0 (8.0–21.0)	0.035	− 0.09	4.5 (1.0–17.0)	11.0 (3.0–22.0)	0.001	− 0.15
Internalizing problems	12.0 (4.3–18.0)	9.0 (5.0–15.0)	0.129	− 0.06	5.0 (1.0–11.8)	4.0 (1.0–10.0)	0.569	− 0.02
Externalizing problems	15.5 (9.3–23.0)	18.0 (11.0–26.0)	0.026	− 0.09	5.5 (1.0–19.8)	14.5 (4.0–27.0)	0.335	− 0.04
Total problems	50.5 (34.5–67.8)	50.0 (34.0–69.0)	0.836	− 0.01	32.5 (15.8–53.0)	48.0 (28.0–67.0)	0.829	− 0.01

Gender comparisons based on TRF showed similar results as CBCL (Table 2). Males with ADHD had more aggressive (*U* = 11,063, *z* = − 3.40, *p* = 0.001, *r* = − 0.15) and delinquent behavior (*U* = 11,696, *z* = − 2.89, *p* = 0.004, *r* = − 0.13). Males also had higher externalizing problem scores while females had higher internalizing scores but both differences did not reach statistical significance. In addition, males were noted to have more thought problems (*U* = 12,641, *z* = − 2.12, *p* = 0.034, *r* = − 0.09), social problems (*U* = 12,482, *z* = − 2.18, *p* = 0.030, *r* = − 0.09) and attention problems, *U* = 10,905, *z* = − 3.53, *p* < 0.001, *r* = − 0.15. Due to heterogeneity of the 12 items in thought problems, individual item was compared between genders using chi-squared tests with Bonferroni correction for multiple comparisons. No significant gender difference was noted in any item.

The effects of age on CBCL syndrome scales, internalizing and externalizing problems were summarized in Table 3. Compared to the younger group, the older group was found to have more internalizing (*U* = 7190, *z* = − 2.63, *p* = 0.009, *r* = − 0.11) and externalizing problems, *U* = 7647, *z* = − 2.15, *p* = 0.032, *r* = − 0.09. The older group had significantly higher scores in withdrawn/depressed problems (*U* = 7532, *z* = − 2.29, *p* = 0.022, *r* = − 0.10), anxious/depressed problems (*U* = 7160, *z* = − 2.67, *p* = 0.008, *r* = − 0.11), attention problems (*U* = 7185, *z* = − 2.64, *p* = 0.008, *r* = − 0.11) and delinquent behavior, *U* = 6804, *z* = − 3.05, *p* = 0.002, *r* = − 0.13.

**Table 3:** CBCL scores by age group

	6–12 years old (*n* = 524)	13–18 years old (*n* = 37)	*P*	Effect size *r*
	*Median (25th–75th quartile)*	*Median (25th–75th quartile)*		
Syndrome scales				
Withdrawn/depressed	2.0 (1.0–5.0)	4.0 (2.0–6.0)	0.022	− 0.10
Somatic complaints	2.0 (0.0–4.0)	2.0 (1.0–4.0)	0.319	− 0.04
Anxious/depressed	4.0 (2.0–8.0)	7.0 (4.5–9.0)	0.008	− 0.11
Social problems	5.0 (3.0–7.0)	6.0 (2.0–9.0)	0.195	− 0.05
Thought problems	2.0 (1.0–4.0)	3.0 (2.0–50)	0.051	− 0.08
Attention problems	10.0 (7.0–12.0)	12.0 (8.5–13.0)	0.008	− 0.11
Delinquent behavior	3.5 (2.0–5.0)	6.0 (3.0–9.0)	0.002	− 0.13
Aggressive behavior	14.0 (8.0–20.0)	17.0 (9.0–23.5)	0.155	− 0.06
Internalizing problems	9.0 (4.25–15.0)	12.0 (8.0–19.0)	0.009	− 0.11
Externalizing problems	17.0 (11.0–25.0)	22.0 (11.0–33.0)	0.032	− 0.09
Total problems	49.0 (34.0–67.0)	63.0 (40.0–76.5)	0.026	− 0.09

In the multiple linear regression model predicting internalizing problem scores, the list of predictors were able to explain 9% of the variance, *F*(9466) = 5.29, *p* < 0.001. Among the predictors, age (*β* = 0.14, *p* = 0.019), financial problems (*β* = 0.09, *p* = 0.048) and physical punishment (*β* = 0.12, *p* = 0.007) were significant predictors (Table 4). In the regression model predicting CBCL externalizing problem score, the list of predictors explained 11% of the variance, *F*(9468) = 6.26, *p* < 0.001. Gender (*β* = 0.17, *p* = 0.043), father’s education level (*β* = − 0.17, *p* = 0.002), psychological aggression between parents (*β* = 0.13, *p* = 0.006) and physical punishment (*β* = 0.18, *p* < 0.001) were significant predictors (Table 4). No significant age × gender interaction was noted in either regression models.

**Table 4:** Summary of multiple linear regressions for variables predicting CBCL internalizing and externalizing scores

Variables	CBCL internalizing score			CBCL externalizing score		
	*B*	*SE*	*β*	*B*	*SE*	*β*
Gender^a^	− 1.38	2.01	− 0.06	5.53	2.72	0.17*
Age	0.49	0.21	0.14*	0.20	0.28	0.04
Gender × age interaction	− 0.52	1.85	− 0.03	− 3.09	2.52	− 0.12
Mother’s education^b^	− 1.22	0.79	− 0.08	− 0.11	1.08	− 0.01
Father’s education^b^	− 1.04	0.79	− 0.07	− 3.43	1.08	− 0.17**
Financial problems^c^	2.46	1.24	0.09*	− 0.01	1.68	0.00
Psychological aggression between spouses^d^	0.59	0.37	0.07	1.41	0.51	0.13**
Physical punishment^e^	1.21	0.45	0.12**	2.38	0.61	0.18**
Family member(s) seen a psychiatrist before^f^	− 1.87	1.01	− 0.08	− 2.12	1.37	− 0.07
*R*^2^		0.09			0.11	
*F*		5.29**			6.26**	

*B* = unstandardized beta coefficient, *SE* = standard error, *β* = standardised beta coefficient

^*^p < 0.05

**p < 0.01

^a^Coded as 0 = female, 1 = male

^b^Coded as 0 = secondary education or below, 1 = post-secondary education or above

^c^Coded as 0 = no financial problems, 1 = experienced financial problems before

^d^Coded as 0 = no psychological aggression between spouse, 1 to 5 = 1 point for the presence of each of the following, yelling, insulting, crying, threatening to injure spouse and refusing to talk

^e^Coded as 0 = no physical punishment in disciplining, 1 = spank or hit with an object, 2 = spank and hit with an object

^f^Coded as 0 = no one in the family has ever seen a psychiatrist, 1 = at least one family member has seen a psychiatrist

## Discussion

This study aimed to elucidate the gender differences in internalizing and externalizing problems in children and adolescents aged six to eighteen with ADHD using parent and teacher reports extracted from a clinical database. Consistent with studies in the West, females with ADHD presented with fewer externalizing problems than their male counterparts. In addition, females presented with more internalizing problems although it was not statistically significant.

### Gender difference in externalizing problems

Gender remained a significant predictor of externalizing problem after controlling for other factors. In contrast to previous clinical studies in Asia where no gender difference was found in externalizing problems, the gender difference noted in our study could be due to two reasons. Firstly, our sample had more females which have increased the statistical power. Secondly, Singaporean parents, similar to most Asian parents, are highly concerned with academic success [34]. Singaporean parents may have a lower threshold to bring their daughters to the clinic when they are facing only academic difficulties or attention deficits, without displaying any behavioral issues.

### Gender difference in internalizing problems

Females with ADHD were reported to have more internalizing problems but the difference did not reach statistical significance. This could be explained by several reasons. Firstly, only parent-reports and teacher-reports were used in assessing the internalizing problems. It has been shown that parent-reports were less sensitive and they tended to underestimate internalizing problems compared to self-reports [35, 36]. Secondly, combining different internalizing syndrome scales into one score may have masked the gender differences in individual syndrome scale. Females were found to be significantly more withdrawn and depressed while no significant gender differences were noted in somatic complaints or anxiety. In a study using self-report questionnaire, males with ADHD reported to experience higher level of anxiety than their female counterparts [37]. The opposite directions of gender differences in individual components of internalizing problems may have resulted in the lack of gender difference in the overall internalizing score.

### Relationship between other factors and internalizing and externalizing problems

In the multiple linear regressions, the regression models could only explain a small percentage of the variance in internalizing and externalizing problems respectively, suggesting that some important predictors were not included in the regression models. After controlling for potential confounders, there was no evidence of a statistically significant association between gender and internalizing problems and only a weak and marginally significant association between gender and externalizing problems. Therefore, the gender differences observed in the univariate analysis could be contributed by gender differences in the relationship between these confounding factors and internalizing or externalizing problems. However, the gender effects of these confounding factors in the ADHD population are unclear. Their potential effects were discussed below.

### Effect of age

Adolescents were found to have more internalizing and externalizing problems than children. After controlling for other variables including gender, age remained a significant predictor of internalizing problems with older age having more internalizing problems. This could be due to the increase in academic demands and higher social expectation of impulse control with increasing age. Inattention, hyperactivity and impulsivity problems may become more impairing in secondary schools, leading to the development of more internalizing problems. Previous literature has shown that an earlier age of onset is associated with more externalizing problems and less internalizing problems [26]. In this study, the age of being diagnosed with ADHD was used as the age of onset was not available. Since most symptoms of ADHD would manifest in early childhood, it is possible that those diagnosed at adolescence may have a longer duration of untreated ADHD leading to a greater severity of impairment. To test this hypothesis, accurate age of onset is needed. However, recall bias is a common obstacle in retrospective research, especially in ADHD studies where symptoms can manifest at a very young age.

### Effect of socioeconomic status

In this study, children and adolescents with ADHD of lower socioeconomic status had more internalizing and externalizing problems. Among studies on socioeconomic status and ADHD prevalence, it remained inconclusive whether low socioeconomic status could be a risk factor for ADHD [6]. Our results suggest a need for intervention targeting internalizing and externalizing problems in ADHD children from families of low socioeconomic status. Raising public awareness of ADHD may facilitate early help seeking from parents of lower education levels.

### Effect of physical punishment and spouse aggression

Consistent with the literature, physical punishment such as caning and spanking was associated with more internalizing and externalizing problems [38]. Based on social learning theory [39], physical punishment can model aggressive behavior of a child and the theory has been confirmed repeatedly in empirical research [38]. Males were found to be more likely than females to receive physical punishment from parents and the effect of physical punishment also differed between genders [40]. Furthermore, self-control was reported to be lower in males disciplined with spanking but no significant difference was noted in females disciplined with spanking [41]. All these evidences suggest that males with ADHD are more likely to receive physical punishment and subsequently perceive poorer self-control and display more aggressive behaviors than females with ADHD. Similar to physical punishment, psychological aggression between parents could have modelled aggressive behaviors in children. While most studies on the effect of physical punishment were done in general population, this study only included participants with ADHD. Future studies should explore whether ADHD could be a moderator on the effect of physical punishment on internalizing and externalizing problems. A recent local study has reported that use of physical punishment was more common in mothers of ADHD children compared to typically developing children but the causal relationship between the use of physical punishment and the children behavioral problems remains unclear [42]. As physical punishment is more widely accepted in the Asian culture, its effect on children development may be different from the West.

### Gender differences in thought problems

Although males with ADHD had more thought problems than females with ADHD according to teacher-reports, no statistically significant gender difference was noted on individual items. Since these items described symptoms from a range of heterogeneous disorders including psychotic disorders and obsessive–compulsive disorder, more comprehensive assessment of these disorders is needed before we can establish their severity and prevalence for gender comparison. Furthermore, the low prevalence of thought problems in children and adolescents necessitates a larger sample for comorbidity study. The gender differences in thought problems observed by teachers in this study may reflect problems in inattention, impulsivity or hyperactivity e.g. “can’t keep his/her mind off certain thoughts”, “twitching”, “deliberately harm self”, “pick nose” and “strange behaviors”.

### Gender differences in social problems

Teachers reported more social problems in males than females with ADHD in the current study. It has been known that both genders with ADHD suffer from social problems including peer rejection and victimization [43–46]. However, little is known about the gender differences in this problem. Males with ADHD may present with more covert aggression and disruptive behavior, resulting in more obvious peer rejection in school. As females and males have different social interaction style, their degree and nature of social dysfunction could be different [47, 48].

## Implications and further studies

Unlike most clinical studies, participants in this study were all newly diagnosed with ADHD and were treatment naïve. Hence, the results were not confounded by interventions. The study has also included the largest number of females with ADHD among studies on gender differences in ADHD in Asia to date. With more female subjects, we were able to capture the gender differences that were not reported previously among young persons with ADHD in Asia. The findings suggest that in the clinical population, males and females with ADHD could present with different comorbidity profiles. Awareness of the gender-specific comorbidity profiles and management addressing the specific needs is warranted.

Although gender differences in ADHD symptoms and comorbidity have been reported more than two decades ago, the causes were not clearly understood. Structural and functional neuroimaging studies have shown numerous gender differences in neuroanatomy and neural network activities [49]. For example, it has been suggested that overproduction of striatal dopamine receptors could be the cause of hyperactivity in male early developmental period [50]. At the molecular level, gender differences in genetics and sex hormones could modulate neuronal development and led to gender differences in neurodevelopmental disorders such as ADHD [51]. However, more research is needed to understand the biological basis of gender differences in ADHD symptoms. Other than biological mechanism, social factors such as socioeconomic status, use of physical punishment and psychological aggression between parents could have an effect on the gender differences in ADHD as suggested in our study. Each of these variables necessitates further research with more well established instruments in order to understand their effect on gender differences.

The lack of externalizing symptoms in females with ADHD may have delayed referral to the professional. A study on barriers to treatment in ADHD has suggested that parents of daughters with high risks of ADHD were less likely to seek help than parents of males with similar problems [52]. Parental perception of ADHD treatment played an important role too as Bussing et al. [52] have suggested a higher perceived stigma for ADHD treatment of daughters than of sons. More research on parental understanding on ADHD, threshold to treatment and stigma is needed in order to improve help seeking in females with ADHD. Furthermore, whether the behavior is labelled as problematic or not could be cultural dependent. Multiethnic study is needed to explore the different cultural thresholds and Singapore, with its multiethnic population, could be a potential study site.

During assessment, clinician should be mindful of the gender differences in the symptoms presented. Gender specific threshold in symptom lists or diagnostic instrument could be a possible solution. However, there is a risk of overdiagnosis in females and it was suggested that the impairment criterion should remain the same for both genders [7]. At the moment, clinicians are still recommended to follow the same diagnostic criteria for both genders. Moreover, further research is needed to explore gender differences in neuropsychological tests in both the ADHD and general population in order to understand whether a gender specific cut off score is necessary. To prevent misdiagnosis, clinician should also consider ADHD as a possible differential diagnosis apart from a mood disorder when they assess females with depressed mood and social withdrawal.

As most studies in female ADHD were based on clinical samples, characteristics of those that were not referred remained largely unknown. Future study using a community sample would help us understand the characteristics of this non-referred ADHD population. A community study would also promote understanding on the inherent gender difference on attention, hyperactivity and impulsivity in the general population. This information would be crucial in development of any gender-specific diagnostic tool or criteria. Apart from clinical criteria, future studies should explore gender differences in commonly used neuropsychological assessment in the ADHD population. This would allow more accurate interpretation of assessment.

### Limitations

There were several limitations in this study. Firstly, the participants were not recruited by nationwide population sampling although they were recruited from the largest child and adolescent psychiatric center in Singapore. Secondly, database was incomplete due to missing data. Thirdly, the database was collected more than ten years ago but it was the largest and most comprehensive of the ADHD clinical population available at the time of the study. Since gender differences in ADHD in externalizing and internalizing problems have been consistently reported over the years, we expect minimal effect of the time gap on the generalizability of our results.

## Conclusion

This study shows that gender differences in comorbidity profiles do exist in Asian ADHD population with males having more externalizing problems than females. Although females were shown to have more internalizing problems than males in the literature, the difference was not significant in our sample. In addition, experiences of physical punishment and lower socioeconomic status were associated with more internalizing and externalizing problems. With other covariates being controlled, age was a better predictor of internalizing problems than gender while gender was a better predictor for externalizing problems than age.

Our results showed that a multi-pronged approach is needed to improve detection of females with ADHD who usually present with fewer externalizing problems. At the parent and school level, we need to improve parental and teachers’ awareness of ADHD and promote help seeking behavior. At the clinician level, we need to re-examine whether gender-specific criteria are needed for diagnosis and whether certain neuropsychological assessments should have gender specific cut off scores.

## Data Availability

The datasets used and/or analyzed during the current study are available from the corresponding author on reasonable request.

## Abbreviations

ADHD: Attention-deficit/hyperactivity disorder
ANOVA: Analysis of variance
CBCL: Child Behavior Checklist
DDP: Department of Developmental Psychiatry
ICD-9-CM: International Classification of Diseases, Ninth Revision, Clinical Modification
IMH: The Institute of Mental Health
TRF: Teacher’s Report Form
